# Data-centric review of multimodal emotion recognition: datasets, feature extraction, data fusion and limitations

**DOI:** 10.3389/frobt.2026.1810478

**Published:** 2026-06-05

**Authors:** Manisha Khanra, Sanjiban Sekhar Roy

**Affiliations:** Vellore Institute of Technology, School of Computer Science and Engineering, Vellore, Tamilnadu, India

**Keywords:** affective computing, data fusion, feature extraction, large language models (LLMs), multimodal datasets, multimodal emotion recognition, self-supervised learning (SSL)

## Abstract

Emotion recognition is a major challenge in affective computing. It enables systems to determine human emotions using multiple modalities, such as text, audio, facial expressions, and physiological data. The field of multimodal emotion recognition (MER) has achieved significant advances in recent years. Present studies tend to focus on single structures and fusion methods only. It provides little interaction between the modalities, input features, and learning models. An overview of MER studies that can be both modality-based and data-driven is provided in the present investigation. We present a methodical assesment of uni-, bi-, and multimodal frameworks, incorporating models to express emotions, feature extraction processes, fusion methods, learning methods, and benchmark datasets. We also provide an individual review approach that integrates performance with textual, acoustic, optical, and physical dimensions. Furthermore, it includes a thorough review for data-centric issues and mitigating approaches. We also consider benchmark multimodal datasets to tackle major problems such as modal diversity, annotating cost, data unavailability, and class imbalance. The MER workflow incorporates novel concepts, including linguistic models based on self-supervised learning. This review identifies opportunities for more robust and sustainable MER frameworks and highlights the remaining research challenges.

## 1 Introduction

### 1.1 Context with motivations

In recent years, emotion recognition (ER) has become a major research problem in affective computing (AC). Emotion recognition makes it easier for machines to improve people’s expertise by allocating sensitive and emotionally intelligent reactions. Scholars from multiple domains (e.g., psychology, pedagogy, neuroscience, cognitive science, and computer science) have progressively extended their observations to AC Song et al. (2019), and it shows the progress of models capable of detecting, translating, and acknowledging human emotions. The capability of machines has become crucial for the concurrent prediction of human emotions, especially in the human-computer interaction (HCI) model Pan et al. (2023). The system recognizes both explicit emotion features, such as facial gestures and verbal tone, and implicit emotions, such as physiological signals, making it a multifaceted challenge. Affective computing can recognize human emotions and intentions. This necessitates the accurate modelling of human emotions using diverse data, such as textual, speech, facial gestures, physiological signals, and body language Egger et al. (2019). To predict human emotions, speech, vocal tone, and facial expressions are often employed, and people can easily hide or shift their emotions. In contrast, physiological signals such as EEG, ECG, and EMG offer a more objective and reliable window into internal affective states Zhang et al. (2020c). These bio-signals are typically instinctive, making them particularly useful in applications that require high emotional fidelity. Affective computing can be used to monitor and diagnose psychiatric disorders. It fixes teaching methods based on student engagement. However, to emphasize conventional unimodal or monomodal emotion detection, a single type of data source is employed, and multiple types of data sources are employed as inputs (e.g., text, auditory, visual, and physiological signals) in cross-modal emotion prediction Chen and Joo (2021). However, these methods fail to identify complex human emotional states. Acoustic features (such as tone, rhythm, and pitch) and the semantic content of spoken words are integrated into speech. Machines can now understand immediate emotional cues from EEG, ECG, facial gestures, and speech. To boost the effectiveness of emotion recognition, textual, visual, and audio data are fused. Although several current studies have clarified the MER framework, but significant analytical limitations remain for the technical data lifecycle.

### 1.2 Difference from existing reviews

Thus, Zhang et al. (2024) primarily work on the emergence of SOTA standards and DL-models (deep learning models) in enhanced, noiseless contexts Zhang et al. (2024). While Pan et al. (2023) propose an extensive guide for the overall HCI process, researchers still prefer fusion approaches across data integrity based on their model-specific approach Pan et al. (2023). Further, a systematic PRISMA-oriented review is provided, and the study primarily concentrates on human-centred applicability (cultural and moral aspects) rather than the technological relevance of data quality Kalateh et al. (2024).

### 1.3 Data-centric approach

Especially, practical issues include sensory noise level, sample size alignment, and missing modality which significantly impact the performance limits of fusion methods cannot be thoroughly explored throughout such studies. Thus, to address these limitations, this study utilizes a completely data-centric framework, considering data limits as the key barrier for MER efficiency. The efficiency of fusion approaches can be substantially affected by such variables. Our study identifies these deficiencies by assuming a completely data-based view and analyzing MER centric on data-based boundaries. Furthermore, it shows LLMs (Large Language Models) and SSL (Self-Supervised Learning) as specific solutions for data limitations compared to simply innovative models approaches. Moreover, recent advances, such as self-supervised learning and large language models, have not yet been systematically positioned within the broader MER workflow. Consequently, researchers lack a unified perspective that connects modality-specific representations, fusion mechanisms, and emerging learning frameworks under real-world data constraints. Recent studies have focused on multimodal sentiment analysis, which integrates various data types to better capture emotional nuances Wani et al. (2021). MER execution and adaptability are increasingly driven by data-related problems despite structural difficulties. Several unresolved problems still exist, such as small datasets, excessive annotations, imbalanced classes, lacking modality, data integrity, data enhancement techniques, source-specific features, inter-subject variation, and multiple-type data fusion, which contribute uniquely to emotion understanding, are often excluded. These issues hinder practical applications and highlight the importance of data-based studies that extend over model precision. This resulted in limited outcomes. Special attention has been paid to data synthesis techniques, which are crucial for overcoming the limitations of scarce multimodal datasets.

### 1.4 Outline of the study

The remaining content of this study is structured as follows: The review strategy and compared context are explained under Section 2. Section 3 presents the backgrounds of emotion recognition over unimodal, bimodal, and multimodal models. Section 4–7 cover the key aspects of MER framework, such emotion models, standardized benchmark datasets, data preprocessing, and feature extraction approaches. Section 8 reviews fusion methods and its related challenges, and Section 9 thoroughly addresses existing obstacles. This study is ultimately concluded in Section 10, which highlights potential future directions in the domain.

### 1.5 Goals and contributions

**FIGURE 1:**
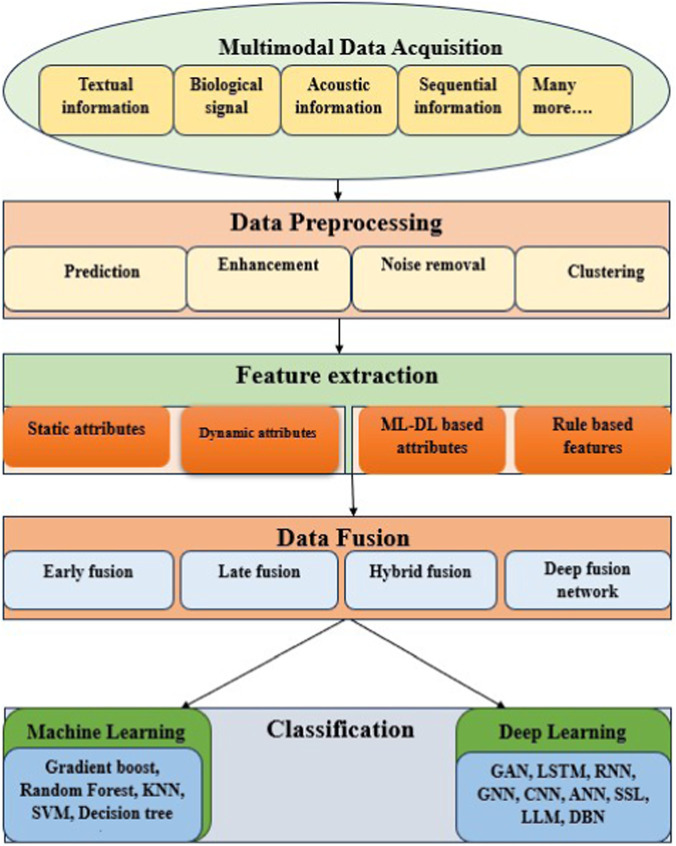
A data-centric framework for MER.

We collected all related articles from IEEE, Elsevier, Springer, and many other popular engineering resources. Below, Table 1 lists the popular words related to our study. This study aims to serve as a complete guide for scholars and practitioners seeking to develop accurate, understandable, and extensive MER methods through modality-driven or data-sensitive studies. Guided by the previously mentioned challenges, including the need for a unified perspective, the key contributions of this study are summarized as follows:

- We provide a data-driven and modality-based classification of MER that systematically organizes previous studies on learning models, data modalities, feature extraction models, and data fusion methods. Therefore, this work presents an orderly structure that connects different MER parts of the system while making it apparent that multiple design choices intersect throughout the entire process, as shown in Figure 1 that adapted and synthesized from (Kalateh et al., 2024). The basic structure is originated from popular frameworks. Still, this study integrates Figure 1 to focus on data-based constraints like sensorial noise or modalities alignment. Such factors tend to be ignored within reviews which focus on conventional models.
- We provide an ordered, cross-modal overview of textual, auditory, visual, and physiological emotions, which led to the given classification. To encourage clear contrasts across modalities in the same conceptual structure, we examine each mode’s distinctive emotional features, relevant model approaches, and fundamental obstacles.
- This study thoroughly focuses on early, late, and hybrid fusion techniques and how they fit within the classification system, along with deep learning and self-supervised representations of features. This study focuses on the development of useful feature fusion methods and highlights instances in which specific methods serve effectively by integrating them under one structure.
- Self-supervised learning and large language models (LLMs) are considered as new learning models within the MER process, rather than individual methods. This highlights its growing impact on future MER models and emphasizes its importance in handling data scarcity using methods such zero-shot transfer learning and synthetic data enhancement, context reasoning, and multidimensional alignment.
- The systematic review of comparison datasets with a focus on limited data or identity-based issues serves as a remarkable feature of this study. Especially, we classify models based on their ability to use bias-control approaches. These comprise approaches for domain adaptability and adversarial learning. This classification illustrates how consistency is preserved in advanced MER models even using limited datasets.
- This review uses a data-driven perspective over all classification phases, extending beyond a model-based review. To convey that data limitations impact modality simulation, feature learning, we explore the challenges of data scaling, annotation expense, lacking modality, and inter-subject and domain-wide biases.
- We integrate key study issues that offer potential futures focused on strong multimodal learning within realistic constraints through the proposed classification. Dynamic combination, subject-specific models, clarity, and data-effective learning are emphasized within these suggestions, and this study also has presented useful suggestions for advancing MER studies.

**TABLE 1:** An abbreviation table for popular words.

Acronyms	Full form
AC	Affective computing
ER	Emotion recognition
MER	Multimodal emotion recognition
LLM	Large language model
HCI	Human-computer interaction
TER	Textual emotion recognition
SER	Speech emotion recognition
FER	Facial emotion recognition
NLP	Natural language processing
SVM	Support vector machine
CNN	Convolutional neural network
GRU	Gated recurrent unit
Bi-GRU	Bidirectional gated recurrent unit
LSTM	Long short-term memory
BiLSTM	Bidirectional LSTM
ELSTM	Enhanced LSTM
ConvLSTM	Convolutional LSTM
IFR-CNN	Improved and faster region-based CNN
RNN	Recurrent neural network
VAVL	Versatile audio-visual learning
DNN	Deep neural network
TVER	Textual visual emotion recognition
ATIA	Audio-text-interactional-attention
MVAN	Multimodal visual-affective network
DMAF	Deep multimodal attention fusion
DCCA	Deep canonical correlation analysis
SSL	Self-supervised learning
M3ER	Multiplicative multimodal emotion recognition
DNTN	Deep noise tracking network
LPS	Log power spectra
IRM	Ideal ratio mask
STFT	Short-time fourier transform
PCA	Principal component analysis
ICA	Independent component analysis
CSP	Common spatial patterns
CQT-MSF	Constant-Q transform based modulation spectral features
HuBERT	Hidden units for BERT
SHAP	Shapley additive exPlanations
API	Application programming interface
GAN	Generative adversarial network
ZCR	Zero-crossing rate
DFT	Discrete fourier transform
ViT	Vision transformer
HDFT	Holistic dynamic frequency transformer
EQCNN	Emotion quantized CNN
BoW	Bag-of-Words
NLU	Natural language understanding
BFTE	BERT-enhanced embeddings
PSD	Power spectral density
DE	Differential entropy
FD-CART	Fractal dimension combined with CART
LDA	Linear discriminant analysis
EMD	Empirical mode decomposition
MSE	Multiscale entropy
GNN	Graph neural networks
FARCER	Fully auto regressive contextual emotion recognition
CBOW	Continuous bag of words
BERT	Bidirectional encoder representations from transformers
RoBERTa	Robustly optimized BERT pretraining approach
DBN	Deep belief network
RF	Random forests
ANN	Artificial neural networks
ELMs	Extreme learning machines
MCB	Multimodal compact bilinear pooling
CBGP	Compact bilinear gated pooling
BDAE	Bimodal deep auto-encoder
FM-SER	Face module-speech emotion recognition
DECN	Dialogical emotion correction network
EEG	Electroencephalography
ECG	Electrocardiography
BVP	Blood volume pulse
EDA	Electro dermal activity
PPS	Photoplethysmography
GSR	Galvanic skin response
DTW	Dynamic time warping
MLP	MultiLayer perceptron
FFN	Fully feedforward network
MoCap	Motion capture
Bimodal DAE	Bimodal deep autoencoder
CART	Communication access real-time translation
CAE	Computer-aided engineering
HFCNN	Hierarchical fusion CNN
HCNN	Hierarchical CNN
FF	Feed forward-network
CRF	Conditional random field
TTRM	Transductive transfer regression model
AGRA	Adversarial graph representation adaptation
PRISMA	Preferred reporting items for systematic reviews and meta-analyses

This study presents an innovative data cycle taxonomies, in addition to previous studies which tend to center on system designs. Specifically, we analyse how data-based drawbacks (such as noise, scarcity, and imbalance) interact with the outcome of unimodal fusion. This presents a way for selecting fusion approaches that consider factors such as the standard of the provided data instead of primarily model uniqueness. Our study addresses the limitations presented in prior studies via an entirely data-centric MER framework to integrate learning models, data features, and modal representations in a new manner.

## 2 Study design and review approach

**FIGURE 2:**
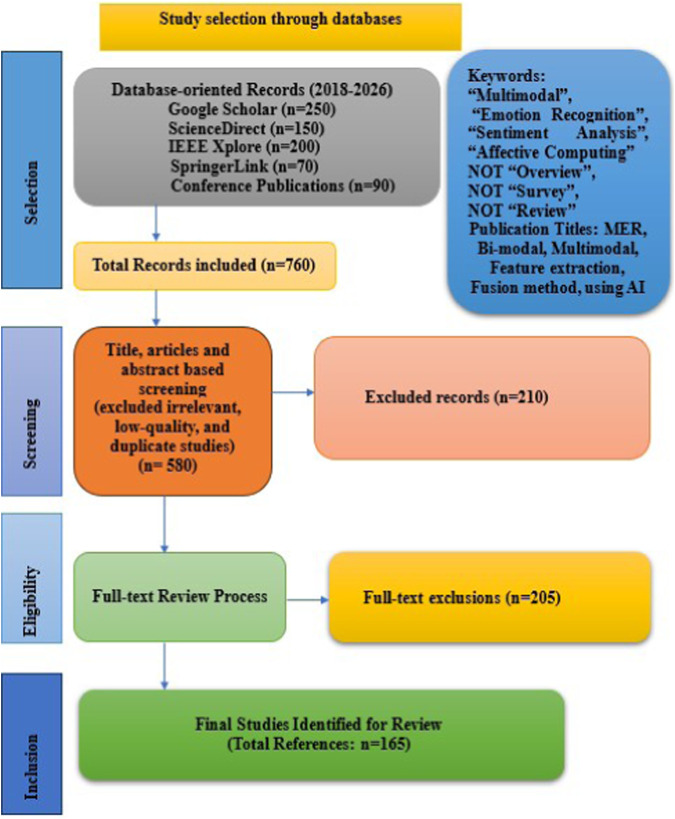
PRISMA structure using keywords, where n denotes the total no. of articles.

**FIGURE 3:**
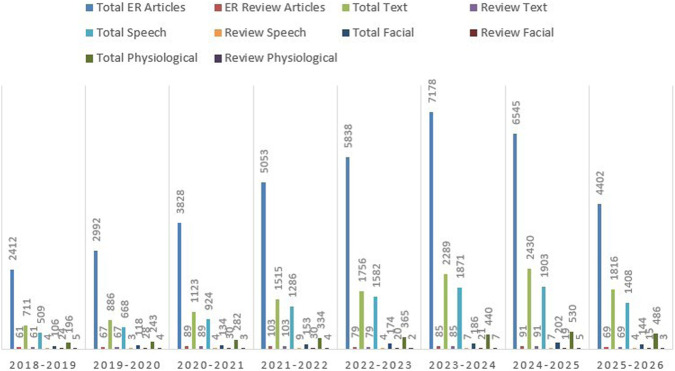
Research papers published on unimodal emotion recognition from 2018 to 2026.

**FIGURE 4:**
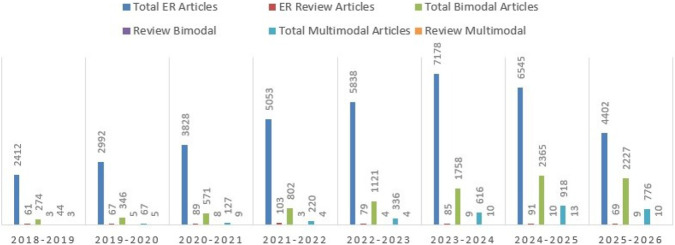
Research papers published on bimodal and multimodal emotion recognition from 2018 from to 2026.

Relevant research on MER was selected from prominent scholarly databases, including Google Scholar, IEEE Xplore, Elsevier (ScienceDirect), SpringerLink and reputed conference proceedings to assure an organized and consistent review process. The keywords like “sentiment analysis,” “emotion recognition,” “affective computing,” “multimodal sentiment analysis,” “multimodal emotion recognition,” “multimodal affective computing,” “feature extraction,” “multimodal fusion,” and” using AI” were employed for the literature review query. Predefined guidelines for inclusion and exclusion requirements, compiled with structured review standards, provided as an outline for the choice of literature. To make sure clarity and consistency, this study selection method aligned to the PRISMA diagram through Figure 2. Figures 3, 4 (idea from Singh and Kapoor (2023)) represent the overall number of articles per year, indicating the overall number of standard reviews, and an overview of the articles related to the specific modality has been shown in these figures. This illustration presents a clear overview of the way standard studies are allocated within the region.

### 2.1 Inclusion criteria

We included the MER-related studies that have already been published in reputed conferences or scholarly journals. We considered the research papers published from 2018 to 2026. Data-centric areas like data preprocessing methods, feature extraction, feature selection approaches, and fusion methods were considered to be addressed in the selected articles. Moreover, each selected paper was required to include at least the following elements: datasets, models, feature extraction, feature selection approaches, fusion methods, and performance metrics.

### 2.2 Exclusion criteria

Publications that exclusively identified single-mode emotion recognition and those that were not available in English were eliminated. Furthermore, studies that duplicated similar results over various datasets without major uniqueness were removed. Unpublished articles, like abstracts, websites, or non-reputed journals, were not included. In addition, the study did not consider any publications that lacked adequate validity, reliability, and a concise scientific method.

### 2.3 Study inclusion and exclusion process

The requirement approach proceeded with a title-specific filtering to exclude ineffective and redundant articles. Then related articles were collected from particular scholarly journals, ensured by abstract-based screening to verify MER compatibility. In the end, based on the predefined inclusion and exclusion standards, full-text reviews have been carried out. This systematic method assured that only useful, high-quality, and scientifically robust studies were considered in the review due to such rigorous methods.

## 3 Background

Previously, emotion recognition analyses centred on single-mode evaluation of text, auditory, visual, and physiological data because of limitations in data accessibility and computational cost. Such single-modal methods built a foundation of emotional computing, but they failed by their capacity to completely represent the intricacy of individual emotions.

### 3.1 Unimodal emotion recognition

Unimodal emotion recognition utilizes one type of input, like text, auditory, facial data, or physiological data. Due to lower computation cost and the accessibility to labelled datasets, those approaches are being thoroughly studied and constitute the foundational base for affective computing studies.

Textual emotion recognition: In the domain of NLP (natural language processing), TER (textual emotion recognition) aims to extract crucial information from written language. However, the lack of non-verbal cues makes it challenging and makes the early method (TER) depend on emotional lexicons and key terms from the datasets. Neural networks and contextually embedded words are both new advances on represent learning, which have significantly improved the efficiency of TER Bharti et al. (2022). Still, there are a few drawbacks to this method, such as redundant or implied reactions, limited multilingual corpora, conflicting tagging strategies, and difficulty in handling humour and context. Therefore, the use of transfer learning, multi-label classification, and transparent deep models is needed for future improvement. For the purpose to identify context and implicit emotional signals, limits promote the synthesis of texts and other modalities.

Speech emotion recognition: Speech emotion recognition (SER) is very important for responsive HCI in area like healthcare and digital assistance. Unimodal methods are computationally effective, but background noise and emotional states are usually hinder their effectiveness. It has given rise to the trend for multimodal approaches utilizing deep learning to synthesize language and speech data. Even after these changes, there are still exist major gaps in the areas of multilingual durability, comprehension, and continuous flexibility Nguyen et al. (2026). The development of transparent, scalable models that are able to maintain accurate results over a range of real-life speech content is essential to address such drawbacks.

Facial emotion recognition: The application of enormous datasets along with advanced models has substantially boosted facial emotion recognition. To identify primary emotional expressions (e.g., happiness, sadness, anger, fear, surprise, and disgust), explanations of facial attributes have been gradually converged by advanced artificial intelligence (AI) Li and Deng (2020). Still, adaptability remains restricted with sensitivity to spatial variability, occlusion, brightness changes, and real-life deployment settings.

Physiological emotion recognition: It unconsciously generated brainwaves, as well as biosignals including ECG, EEG, and EMG, are employed via physiological signals for emotion classification to track internal emotional states. Self- and cross-domain deep learning models using ECG and EEG signals have achieved high accuracy and noise resilience in emotion recognition Li et al. (2021), Mahmoud et al. (2023). However, real-life applications and scalability remain limited by challenges, such as inter-subject variability and limited labelled datasets.

Such single-mode approaches developed an adequate basis for emotions, yet there are certain limits. These limitations render it challenging for individuals to express the intricacy of human emotions. As an outcome, a transformation to increasingly interrelated systems are needed. Such gaps are overcome by MER, that integrates multiple datasets. Still, a new dimension of intricacy is provided by this fusion. Additionally, modality-based drawbacks and data lifecycle handling raise obstacles. The primary MER architecture is presented the subsection that proceeds to attempt to systematically determine whether these different data are arranged and employed. It begins by using basic representations of emotion. Further, it focuses on benchmark data sets that constitute the conceptual basis for multimodal approach.

### 3.2 Bimodal emotion recognition

Bimodal emotion recognition integrates two different types of data to enhance reliability over single-modal models. Bimodal methods are still vulnerable to imbalances in data and synchronization issues, but can reduce a few of the limitations of unimodal models.

Audio-visual emotion recognition: Audio-visual emotion recognition adds facial and speech cues; however, it still suffers from noisy, sparse, or skewed real-world data. In addition, the adaptable Audio-Visual Learning (VAVL) framework enables unimodal and multimodal emotion recognition with shared layers and reconstruction objectives and achieves strong results on CMU-MOSEI, MSP-IMPROV, and CREMA-D Goncalves et al. (2024). Many systems still need complete modalities, which make unified transformer-based models like VAVL essential. Audio-visual models still remain subject to periodic imbalance, along with data-driven imbalance, with improved performance.

Audio-textual emotion recognition: Recent research combines audio and text data to improve emotion recognition, along with text that provides semantic–syntactic cues and speech that carries rhythm, tone, and prosodic information. A model named ATIA integrates acoustic–textual cues using ArcFace loss, and MER-HAN uses hierarchical attention and surpassed prior models on MELD and IEMOCAP Tang et al. (2022), Zhang et al. (2023). There are other issues, such as modality imbalance, synchronisation errors, and computational cost. Development of attention-based and transformer-based fusion methods is inspired by these limitations.

Text-Visual emotion recognition: It strives to more accurately detect human emotions by incorporating textual and visual data, and methods like DMAF and MVAN improve aligning and fusing between these Huang et al. (2019). The invention is additionally added with an extensive dataset of 190,000 images and text pairing that give significant emotional details Yang et al. (2020). For better emotion detection, memory-based models also help to detect regular multimodal exchanges Xu et al. (2018). A primary challenge persists generability over channels with poor online information.

EEG-Facial emotion recognition: Facial expressions provide explicit emotive signs, while EEG data contain subtle emotional states, and fusing them boosts emotion recognition. A dual-phase model fusing 1D-CNN and ResNet50 with DCCA revealed significant integration of EEG and facial cues on MAHNOB-HCI and DEAP datasets Muhammad et al. (2023). A late-fusion approach based on Dempster-Shafer theory boosted reliability over noisy and modal diversity on MAHNOB-HCI, SEED, and their private dataset Lian et al. (2025). Still, limitations like insufficient integration, noisy EEG signals, imbalanced data, and high computational costs remain, hindering real-time usage, requiring present work to insist upon attention-based fusion, flexible models, and cross-domain transfer learning. Real-time durability and reliability problems are not resolved for real-world applications.

### 3.3 Multimodal emotion recognition

MER collectively combines various types of data to collect additional and indirect emotive details. To enhance performance, textual, acoustic, and physiological data are fused, but this poses issues with data alignment, fusion method, and missing modality. In comparison to earlier approaches, new MER approaches focus on data-effective learning, representations aligning, and adaptive fusion to cope with real-life insufficient data Mittal et al. (2020), Le et al. (2023). Such developments indicate an advance into MER models that can be adaptable, flexible, and human-oriented. Table 2 illustrates the background of MER.

**TABLE 2:** Summary of related studies based on modality, methods, benefits, and limitations.

Study (Year)	Modality type	Modality/Ies	Methods	Benefits	Limitations
Aftab et al. (2022)	Unimodal	Speech	Compact fully CNN and simultaneous paths utilizing MFCCs	Compressed, data-scarce, high reliability, real-time consistent	May underachieve with highly dimensional or unclean data
Chowdary et al. (2023)	Unimodal	Facial images	Transfer learning (VGG19, MobileNet, ResNet50, inception V3)	Up to 96% recognition rate; training effective	Small dataset range, gray-level images
Olusegun et al. (2023)	Unimodal	Text	LSTM, BiLSTM, CNN, CLSTM; SMOTE; NRCLex	96% accuracy (CNN), cross-lingual, synchronous	Biased datasets, informal tweets
Jiang et al. (2021)	Bimodal	EEG and face data	CNN-LSTM, LSTM	Acquires indirect features	Unimodal inferior
Stappen et al. (2021)	Bimodal	Facial and textual data	Sentic fusion, facial classifier	Fuses attributes effectively	Hard to acquire quick facial cues
Xing et al. (2023)	Bimodal	Multiple biosignal data	InfoCon	Matches attributes well	Requires mixed training
Islam et al. (2024)	Bimodal	Video and physiological data	BiLSTM, attention mechanism, CNN	Helpful for health services	Degraded input quality fails to recognize facial expression
Lee et al. (2021)	Multimodal	Textual, acoustic, and vision data	Attention fusion and BERT	Fuses signals effectively	Hierarchical fusion
Jia et al. (2022)	Multimodal	Speech, video, and MoCAP data	Decision-level fusion (dual spectrum, CNN, GRU, 3D CNN, Bi-LSTM, attention)	Records temporal-frequency cues; reveals facial movement; acquires activity-based emotion events; enhances reliability by 9%	Limited on original emotion; requires face integrity; some attributes decrease accuracy; some attributes decrease accuracy; requires optimization of modality weights
Cheng et al. (2023)	Multimodal	Textual, acoustic, and visual data	Attentional temporal convolutional network (ATCN) + multi-layer feature fusion (MFF)	High performance, effectiveness, and contextual sensitivity	Rank deficiency, data overlapping
Zhao et al. (2021)	Multimodal	EEG, GSR, EOG, ECG, temperature, respiration belt	Multiscale CNN + biologically inspired fusion	Superior accuracy, adaptability, effective fusion	High computational cost, less flexible and handcrafted attribute dependency
Li et al. (2025)	Multimodal	EEG and external biosignals	CNN, LSTM, feature fusion	High reliability, records discriminative attributes	Challenging, sensor-specific, DEAP-only

Still, the basic abilities of ER (emotion recognition) have already been developed by such foundation models (unimodal, bimodal, and multimodal). Data-related problems usually hamper its real-world use. These obstacles comprise modalities synchronization difficulties, noise, and data imbalances. An organized computational model is essential to evaluate the complete data flow so as to get over a conceptual knowledge. As a result, the basic parts of this architecture are outlined thoroughly (section4 to section7). As an outcome, the key elements of this architecture are presented comprehensively from Section 4 to Section 7, begin with the fundamental emotional representations as well as the benchmark data sets that constitute the technical base of existing MER frameworks.

## 4 Emotion model with methods

Emotion models are the basic framework of affective computing (AC) and enable structured representations for computing devices to understand, learn, and determine human emotions.

### 4.1 Model classification

**FIGURE 5:**
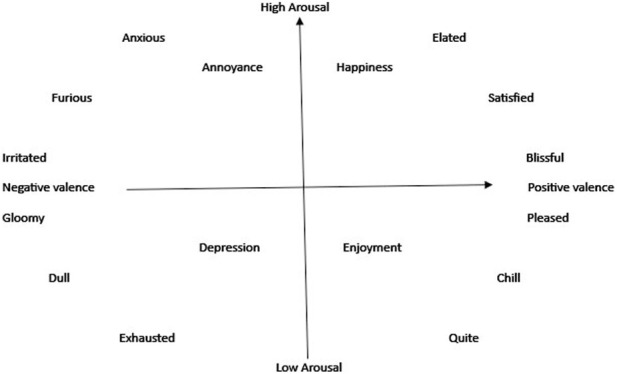
A basic VAD model.

**FIGURE 6:**
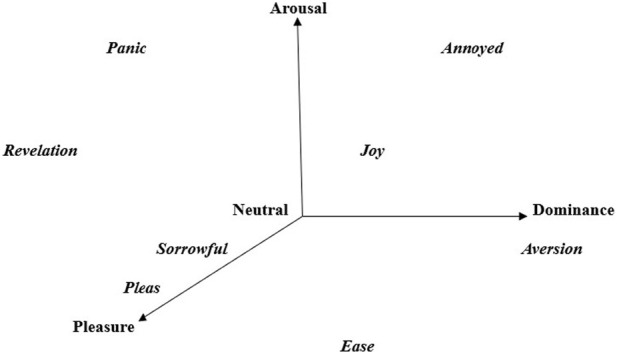
A basic PAD model.

Human emotion modelling comprises two main architectures: one is a discrete model, and the other is a dimensional model. According to Ekman, the discrete model identifies six universal emotions, such as anger, disgust, fear, sadness, happiness, and surprise Ekman and Friesen (1971). The discrete model continues to be used in ER standards for its clarity and cognitive base. In contrast, dimensional models are more suitable for modelling emotional patterns of spoken words, gestures, and physiological signal data due to their continuous and precise nature. Still, poor annotation and limited accessibility in future decision-making remain prevalent issues using multiple methods. The most commonly used models are the VAD (valence-arousal-dominance) model and the PAD (pleasure-arousal-dominance) model. The VAD model locates emotions by positivity (valence) and intensity (arousal), and the PAD model includes a dominance dimension that reflects emotion control Mehrabian and Russell (1974). Figures 5, 6 illustrate a basic VAD and PAD emotion model which are adapted and synthesized from Khare et al. (2024) and Pan et al. (2023).

### 4.2 Emotion recognition process

Emotion detection in multimodal systems follows a structured pipeline. While hybrid and late fusion methods enhance computational cost and enhancing early fusion, and reliability, frameworks struggle to efficiently combine different feature fields. To better address missing modalities and real-world disruption, recent studies emphasize attention-based fusion, self-supervised learning, and transformer structures. It starts with data collection, preprocessing, feature extraction, data fusion, and classification. This fusion method enhances accuracy by leveraging the strengths of each modality. New MER systems prioritize context-sensitive representation learning to boost generality over topics and interface settings. We have presented existing reviews of multimodal emotion recognition in Table 3.

**TABLE 3:** Existing reviews for multimodal emotion recognition.

Publications	Dataset used	Data preprocessing	Combination of multiple data types
Zhao et al. (2019)	✓	×	A + V, A + V + T
Jiang et al. (2020b)	✓	×	A + V, V + T, A + V + T, V + P
Shoumy et al. (2020)	✓	✓	A + V, V + T + P, V + P
Abdullah et al. (2021)	✓	×	A + T, I + T, F + P
Rouast et al. (2019)	✓	✓	A + V + P
Wang et al. (2022b)	✓	×	V + A, T + A, V + A + T, MP, PP
Ahmed et al. (2023)	✓	×	S + A, I + V + F
Hosseini et al. (2024)	✓	✓	T + A, V + A, V + T, T + A + V
Kalateh et al. (2024)	✓	✓	A + V, A + P, V + P, A + V + P
Zhang et al. (2024)	✓	✓	A + V, A + V + T
Abbas et al. (2025)	✓	✓	A + V + T
Li et al. (2026)	✓	×	A + V + T
Our study	✓	✓	A + V, A + T, V + T, A + V + T, MP, OF

Descriptor: A, audio; V = visual; T, textual; I, image; F, face; P, physiology; MP, multiple physiological signals; BM, body movement; PP, Physical-Physiological; OF, others fusion.

## 5 Emotion dataset from multiple data sources

A multimodal emotion database combines two or more data sources across acted and spontaneous datasets. Acted data offer clear but less authentic expressions, whereas spontaneous data provide natural yet noisy affective cues from recordings and online media. A combination of such data provides essential resources to facilitate real-world MER studies. By combining both direct and indirect emotions, multimodal datasets provide more reliability and deeper emotional representation than unimodal datasets. All of these advantages, generating large and comprehensive multimodal emotion databases, remain costly and challenging. Table 4 presents a multimodal dataset with its modality, subjects, emotional description, limitations of dataset usage, annotation consistency, biases, and the distribution of dataset class. Class imbalance is an ongoing issue that causes learning models to skew toward majority emotion groups and perform poorly on minor emotions due to a lack of uncommon emotions.

**TABLE 4:** Popular multimodal datasets for ER.

References	Dataset	Modality	Stimuli with subjects	Emotion description	Usage limitations	Annotation consistency	Biases	Class distribution
Livingstone and Russo (2018)	RAVDESS	Acoustic and visual	44 melodies, 60 monologues, 24 subjects	Happiness, calmness, sadness, disgust, anger, fear, surprise, neutral	Open access	High	Lab-controlled	Balanced
Perepelkina et al. (2018)	RAMAS	Acoustic, visual, body pose, bio signals	7 h video samples, 10 subjects	Anger, scare, disgust, happiness, surprise, sadness	Limited access	Expert-labelled	Linguistic and cultural (Russian)	Balanced
Zadeh et al. (2018b)	CMU-MOSEI	Acoustic, visual, text	3,229 videos with 22,676 speech units, ∼ 1,000 subjects	Anger, sadness, disgust, fear, surprise, happiness	Limited access	Crowd-sourced labels	Noise and gender	Imbalanced
Zadeh et al. (2018a)	MOUD	Text, auditory, video	80 feedback videos, 101 subjects (Spanish)	Positive, negative, neutral	Limited access	Unified labels	Linguistic biases (Spanish language)	Balanced
Hazarika et al. (2018b), Hazarika et al. (2021), Majumder et al. (2019)	IEMOCAP	Auditory, video, text	Nearly 12 h data, 10 subjects	Happiness, sadness, anger, frustration, neutral, valence, activation, dominance	Limited access	Moderate labels	Socio-demographic	Imbalanced
Hazarika et al. (2018a)	SEMAINE	Audio and video	959 verbal interactions, 150 subjects	Five affective aspects and 27 classes	Limited access	High	Interpersonal bias	Imbalanced
Nakisa et al. (2018), Li et al. (2019)	MAHNOB-HCI	Auditory, video, bio signals, eye movement	540 AV recordings, 532 physiological samples, 27 subjects	Valence, arousal, dominance, forecastability, emotional cues	Limited access	Hybrid	Lab-controlled	Imbalanced
Ma et al. (2019a), Liu et al. (2020), Ren et al. (2019)	DEAP	Video, biosignals	32 biosignals, 22 videos, 32 subjects	Valence, arousal, dominance	Limited access	User labels	Lab-controlled	Imbalanced
Sharma et al. (2019)	CASE	ECG, EMG, BVP, EDA/GSR, respiration, skin temperature	Videos, 30 subjects	Arousal and valence (dimensional)	Open access	High	Limited demographic and lab-controlled	Targeted balanced (A-V)
Cornejo and Pedrini (2019), Singh et al. (2019), Pan et al. (2020)	BAUM-1	Auditory, video	1,184 AV recordings, 31 subjects	Six primary emotions	Limited access	Expert with bimodal sync	Stimulus, subjective, cultural (Turkish), lab-controlled	Imbalanced
Yu et al. (2020)	CH-SIMS	Auditory, optical, text	2,281 video samples	Positive, neutral, negative, weakly positive, weakly negative	Open access	Uni- and multimodal labeled	Gender, linguistic, and cultural (Chinese)	Imbalanced
Chen et al. (2021a)	HEU	Auditory, visual, body pose	19,004 videos, 9,951 subjects	Anger, fear, bored, disappointed, confused, disgust, neutral, happy, surprise, sad	Limited access	Attention-based fusion	Linguistic and cross-cultural	Imbalanced
Praveen et al. (2021)	RECOLA	Auditory, video, bio signals	27 AV samples, 46 subjects	Arousal-valence dimensions	Limited access	Continueous with post-processsed	Limited diversity, age-gender and linguistic (French)	Imbalanced
Sharma et al. (2021)	VGAF	Acoustic and videos	4,183 YouTube videos (comprises 587,364 clips); digital, education, and conflicts of people’s opinion (subjects)	Valence: Group expressions on positive, negative, and neutral	Limited access	Expert-guided	High-diversity and valence-based	Imbalanced
Waligora et al. (2024)	Aff-Wild2, BioVid	Facial, speech, bio signals	564 videos, 87 subjects	Arousal-valence and pain intensity	Limited access	Affective with pain-labels	Balanced demographic, mixed participants and social-media variability	Imbalanced
Jiang et al. (2024)	SEED-VII	EEG, eye gaze	80 different video clips; 20 subjects (80 episodes)	6 primary emotions (happy, sad, anxiety, distaste, wondering, and irritation) with neutral emotions	Limited access	Continuous and self-annotated	Small datasets, bias-controlled	Balanced

This variation makes the model harder and reduces the trustworthiness of commonly employed evaluation metrics such as total precision. For thorough evaluation and data setup, ER methods are often organized within bimodal and basic multimodal settings that reflect the varieties of aspects used.

A crucial difference between controllable dependability and environmental reliability is illustrated through an extensive review of the benchmark datasets presented in Table 4. Though there are highly consistent annotations, whereas initial datasets such RECOLA as well as DEAP contain limited samples with demographic limitations. In the other aspect, newer “in-the-wild” datasets that include HEU emotion data as well as CH-SIMS present the scale of data essential for traditional deep learning. These still, result in substantial differences in class and distortion related to identities. Based on synthesis approach, annotating variance over dimensions than dataset size is the key issue of MER. As an example, cross-corpora assessment becomes more complex through the transition from distinct classification labels to constant dimension labels (V-A). This transition provides the adoptability of SSL to address such inconsistencies.

## 6 Data preprocessing

Data pre-processing is a crucial step in MER that helps to remove noise like whitespace, symbols, and numbers to enhance clarity and accuracy. Each modality, like facial images, voice signals, and body-relevant signals, passes through separate pre-processing to improve feature extraction and emotion interpretation. The preprocessing method is extremely contextual and model-specific because, particularly within deep learning-based MER models, insufficient processing can result in the removal of emotion-related data.

### 6.1 Preprocessing of textual data

The text preprocessing technique is an important step of emotion recognition from the textual context. It aims to filter raw data for effective feature extraction and classification. The normalization of textual data by stemming, tokenization, or lemmatization, which reduces lexical variance and preserves emotive meaning, serves as an essential step for this method Palomino and Aider (2022). This includes handling punctuation, spacing inconsistencies, and emotive features such as punctuation marks, numbers, and symbols. These features are often removed, as they commonly carry minimal semantic value, and they can interfere with text analysis algorithms. Still, new studies show numerical ratings, punctuation patterns, and repeating symbols can represent hidden emotional depth. Therefore, the tasks and features of the dataset must guide the elimination of such elements. Emojis and emoticons are preserved or even specially processed. They serve as key indicators of emotional states like happiness, sadness, or anger to enhance emotion accuracy. Emoticons can be connected to emotional representation and processed as distinct emotional signals within current MER models, boosting their capacity to define emotions. In addition, to reduce noise and improve the sentiment model’s accuracy, preprocessing includes lowercasing text, expanding abbreviations, and removing irrelevant or incomplete sentences Amanat et al. (2022). Still, transform-based models, which can acquire context and morphological differences using initial or poorly transformed text, use these preprocessing methods quite severely. These steps enhance emotional context representation in MER. Audio data needs the same improvement method, although with text preparation, the signal-level shifts require a focus on recording context, periodic distortion, or speech variation, as discussed in the following section.

### 6.2 Preprocessing of audio data

Emotion can also be expressed through speech or vocal tone, which comes under acoustic cues. The pre-processing of acoustic signals is necessary in speech emotion recognition (SER) due to the inherently inconsistent nature of speech signals. Differences in speaking speed, channel conditions, noise levels, and speaker-specific features complicate effective emotion detection using raw audio data. To manage this, signals are split into short frames (typically 20–30 m), where speech properties can be considered as quasi-stationary. Window methods include Hamming, Hanning, and rectangular windows. These window methods reduce spectral leakage and retain natural speech features and time consistency for short-time evaluation. A noise-adaptive variational autoencoder (VAE) with statistical feedback was presented for SER Bando et al. (2020). This method integrates adaptive noise modelling and generates models to enhance robustness for noisy and hidden audio environments. In this method, a generative audio model and noise-sensitive adaptation are jointly combined to improve the performance of classical models under unknown scenarios. Noise reduction techniques are applied to minimise the background noise and maximise signal clarity before feature extraction. However, high noise reduction may affect prosodic signals, which may be emotionally essential and need appropriate parameter selection. The consistency of voice segments is a significant aspect of acoustic data preprocessing. It eliminates voiceless and silent regions by using zero-crossing rate and autocorrelation, which are known as classical methods, and makes it rich in emotional and linguistic content Ma et al. (2019b). This step ensures the subsequent representations dominate with emotionally informative sections. Deep Noise Tracking Network for single-channel speech and BiLSTM-based models using LPS (Log Power Spectra) and IRM (Ideal Ratio Mask) are some advanced deep learning models Nie et al. (2018). Such models also learn feature enhancement with noise reduction in a data-driven manner, compared to conventional preprocessing methods. These improve the quality of acoustic data, though performance still depends on environmental conditions for multi-channel enhancement Cui et al. (2021).

### 6.3 Preprocessing of facial data

Facial data preprocessing is a critical component of facial expression recognition (FER). It includes face detection, alignment, and normalisation to reduce variations caused by lighting, pose, and background noise Kumar and Rajagopal (2019). Sequence-based methods capture subtle emotion changes over time but require higher computational resources and are sensitive to noise Sun et al. (2019). Normalising face alignment thereby enables consistent facial feature analysis across frames and subjects. Recently, models like cascaded CNNs and feature-based CNNs have been employed for more accurate facial emotion recognition and feature representation Guo et al. (2020). Improvements in classification performance for age, gender, and emotion recognition have been reported through enhanced CNN variants Khattak et al. (2022). The accuracy of emotion classification has increased by integrating the PAD (Pleasure–Arousal–Dominance) model with a traditional AlexNet CNN, which highlights the impact of emotional model-based facial data enhancement Tang et al. (2020). However, the robustness of these sequential facial preprocessing methods for realistic FER systems can be restricted due to their extreme susceptibility to light changes, occlusions, and subject-specific variability.

### 6.4 Preprocessing of physiological data

The preprocessing of physiological signals plays a critical role in accurately detecting emotions. PPG (photoplethysmography) and EMG (electromyography) are raw physiological signals that usually include motion artifacts, sensor-related noise, and baseline drift. Segmentation, denoising, and normalization are standard preprocessing steps that help to retain relevant signal patterns Han et al. (2023). The Short-Time Fourier Transform (STFT) is commonly applied to analyze non-stationary signals such as EEG and EMG. This change retains spectral traits and temporal dynamics, which are essential to recognize emotional patterns. Band-specific filtering utilizes high-pass, low-pass, or Butterworth bandpass filters. It helps to distinguish frequency ranges by associating them with different emotional processes (e.g., delta (0.5–4 Hz), theta (4–8 Hz), alpha (8–13 Hz), beta (13–30 Hz), and gamma (30–43 Hz) bands Houssein et al. (2022). PCA (Principal Component Analysis), ICA (Independent Component Analysis), and CSP (Common Spatial Patterns) techniques improve signal quality by removing unwanted components and reducing dimensionality. High-dimensional physical data are minimized in size via PCA, which provides a number of linearly independent variables referred to as principal components. These approaches minimize computational cost and preserve the maximum variability of physiological signals. In an effort to distinguish emotion-based neural brain function from visual or muscular distortions in the EEG dataset, ICA is also utilized to split multidimensional signals into additional subcomponents. In making sure that the data input for the fusion level is simultaneously concise and noiseless, these approaches collaborate effectively to prevent models from overfitting on irrelevant physiological data. These methods make the dataset more suitable for emotion identification. Significant cross-subject variation, changes in sensory position, and sensitivities toward motion-based errors render physiological data preprocessing hard. These variables may limit the applicability of emotion recognition models over individuals and record scenarios.

## 7 Emotional feature extraction

In this emotional feature extraction phase, the multimodal preprocessed data are used to be transformed into discriminative features that retain emotions-relevant info during modal-specific preprocessing. These features provide the base of both single- and multimodal emotion recognition model. Attribute extraction is also known as feature extraction. The primary objective of attribute extraction is to extract raw multiple input into discriminant models to recognize emotion. Emotion can be inferred employing both unimodal and cross-modal scenarios. Unimodal approaches are dependent on one type of data source, and the cross-modal approach incorporates various types of data sources to maximize the performance and robustness of ER systems. Under both scenarios, it is necessary to extract emotional attributes for separating significant patterns and detecting emotion more precisely. The classification of emotions and unstructured data interacts through feature extraction. In text extraction, deep contextual embeddings that preserve the background of words within a text are derived by conventional models like BERT. It helps the model to interpret nuanced emotions such as humour or sarcastic. MFCCs, which precisely capture prosodic attributes such as tones and rhythms that support arousal, are extracted from the acoustic features and used to define the short-term power spectrum of the audio. CNNs in the visual domain identify subtle gestures that correspond to different emotions by extracting spatial information from facial regions. The most distinctive emotions persist before the fusion level due to modality-based extraction. Like, attribute extraction, efficient emotion detection models need a reduction of dimensionality and attribute selection. By excluding irrelevant or noisy data and attribute selection (feature selection), it improves the performance and clarity of the model. In addition, by extending attributes in less-dimensional spaces and retaining significant emotional data, dimensionality reduction methods are employed to process highly complex multimodal data. To enhance adaptability over datasets and improve computational effectiveness, such methods are important. Identifying the most emotion-relevant features from the feature collection needs feature selection. The model can gradually identify a specific set of attributes -for example certain EEG spectrum bands or significant facial markers to enhance the accuracy rate of classification via evolutionary computing and genetic algorithms. These procedures primarily minimize the dimension of the fusion vectors as well as maximizes the readability of the model.

### 7.1 Textual features

Textual features have a crucial role in text-oriented emotion detection. Traditionally, approaches like bag-of-words and rule-based approaches are utilized to identify these features. These methods offer interpretable but shallow representations. Hybrid models combine word2vec, syntactic features, and autoencoders to improve semantic depth Su et al. (2018). Pretrained industrial APIs support real-world deployment, like IBM Watson’s Natural Language Understanding (NLU) API to extract emotional vectors from Twitter for abuse detection Benrouba and Boudour (2023). Deep learning models, including CNN-CBOW, hierarchical CNN-LSTM, and ELSTM with empathy-attention mechanisms, are used to improve emotional sensitivity and contextual modelling. Further performance was enhanced using BERT embeddings with CNN-LSTM pipelines [Liu (2020)], Wang et al. (2019), Huang et al. (2021), Kumar and Raman (2022). However, textual emotion models are affected by domain-specific language, irony, linguistic ambiguity, and contextual sparsity. Such factors can reduce the adaptability of real-world ER circumstances and hinder generality over databases. Attribute selection methods, including embedded filtering and attention mechanisms are utilized to preserve contextually relevant words and reduce redundant data, in addition to boost efficiency. Moreover, high-dimensional textual attribute spaces are controlled, and computational effectiveness is enhanced through dimensionality reduction methods like embedded compression and autoencoder-based models.

### 7.2 Audio features

Self-supervised and deep spectral models, which effectively represent spatial as well as affective variance, have substituted manual prosodic traits in audio attribute extraction. In addition to textual content, speech signals provide a rich source of emotional information by combining spectral, temporal cues, and prosodic elements for ER. Traditional methods for SER (speech emotion recognition) depend on manually produced acoustic traits, including speech speed, pitch, and energy. Mel-frequency cepstral coefficients (MFCCs) and zero-crossing rate (ZCR) approaches capture the rhythm- and intensity-related patterns in speech. Time frequency models achieved by transforms like STFT boost more robustness over signal variability and noise Chowdhury et al. (2025). Deep learning methods, which contain 2D CNN (two-dimensional convolutional neural network) trained on DFT-based spectrograms, were developed and shown to have superior performance over several kinds of speech emotion databases Singh and Goel (2023). Sequence-sensitive approaches employing speech rate along with rhythm data integrated with LSTM models enable enhanced temporal emotion prediction Guo et al. (2022), Yang et al. (2024). Domain adaptation and transfer learning models, such as DAN (domain adversarial network), have been studied to tackle speaker with domain variability Abdelwahab and Busso (2018). Recently, self-supervised models such as HuBERT have proven models can employ all global and local attention mechanisms to generate emotion models through unlabelled and noisy speech Jin et al. (2025). Still, inter-corpus generalization is restricted because audio features remain affected by speech variance, recording context, and linguistic speech patterns. In acoustic data processing, attribute selection seeks to remove redundant data and identify highly distinctive speech aspects, including speech tone and spectrum changes. High-dimensional spectrum and audio features, like PCA (Principal Component Analysis) as well as deep autoencoders, that enhance durability and reduce computational cost.

### 7.3 Facial features

Emotion recognition primarily depends on facial expression. The key components of FER (facial expression recognition) are facial landmarks, geometrical distances, and textures. Previous FER methods depended on manually generated or rule-based aspects, which performed effectively in controlled environments. These aspects were typically split into appearance-based, geometric, and motion-based models [Hassan et al. (2019b)], Li and Deng (2020). However, the temporal progression of facial expression is not completely captured by any static models. Such dynamic approaches for feature extraction have emerged for better recognizing patterns of temporal emotions by simulating face variations over time via image sequencing Parveen et al. (2021). Due to the invention of deep learning, CNNs are now widely utilized for developing complex facial models using greyscale and coloured images. These have removed the requirement for traditional feature engineering Verma and Verma (2020). As pretrained models such as AlexNet, ResNet, and VGG improve on task-dependent facial data, transfer learning continues to enhance FER performance within restricted data contexts Meena et al. (2024). Adaptive frameworks, including TTRM and AGRA, are being invented to tackle cross-domain variance Zong et al. (2019), Chen et al. (2021b). Real-time FER deployments are rendered feasible with lightweight frameworks, like MobileNet and EfficientNet, which offer superior performance with less computational cost Haq et al. (2024). With the aim to enhance the model robustness under uncontrolled settings, GANs (Generative adversarial networks) were also employed for data enrichment and identity normalization Zhang et al. (2021a). With these advancements, FER systems remain affected by changes in illumination, head position, occlusions, and subject-specific expressions. All of these persist to limit generalization in reality. Attribute selection is utilized in FER to identify significant facial attributes like the facial expression, foreheads, and gaze, often using attention mechanisms. Thus, to ensure efficient analysis while retaining essential spatio-temporal affective trends, dimensionality reduction methods contribute to reduce highly dimensional facial images captured by traditional deep networks.

### 7.4 Physiological features

Physiological signals provide a hidden and robust method for emotion detection, while they represent spontaneous responses regulated by both the brain and the external nervous system. EEG (electroencephalography) has a direct relationship with the brainwave activity that generates emotional responses. It receives an enormous amount of attention across these signals. Time-domain variables (such as Hjorth parameters and fractional dimension), time-frequency (like wavelets and higher-order spectral), and frequency-domain (like, PSD, DE) representations are examples of conventional EEG-based emotional features. These models were generated by wavelet-based approaches Houssein et al. (2022). To boost the performance of emotional classification, deep learning models are increasingly utilizing different EEG models like CWT (continuous wavelet transform) features, along with neural networks like GoogLeNet Aslan (2022). Further recent study addresses spatial and structural representations via graph-based learning, where inter-channel connections and cortical dependencies are identified by electrode-specific data defined into 2D architectures Pham et al. (2025), Song et al. (2021). Additionally, attention-based and transfer learning models have been studied to enhance generality over different recording environments and minimize inter-subject variance Du et al. (2020). Considering such advances, physiological features still remain affected by noise, sensor context, and subject-specific variability, which hampers their scalability in real-life emotion detection models. Identifying the most effective channel and frequencies in biological signals is essential for their significant complexities and noise. Compressed and relevant models are derived via dimension reduction methods PCA, and deep-learning-driven encoders that increase classification accuracy and minimize subject-specific variance.

Future efficacy is mostly modality-specific, based on a comparative review of existing literature. As an illustration, MFCCs persist as a benchmark of acoustic due to its minimal computation cost. Yet, due to Log-Mel Spectrograms are more effective for identifying time-spectral subtlety, it frequently generates better accuracy within deep learning models. In text-based extraction, conventional Word2Vec frameworks usually fail to identify emotions for limited social media content. However, Transformer-based embedding (BERT/RoBERTa) execute those by using bi-directional perspective. Geometric based aspects are additionally adaptable to shifts during face analysis. In opposite, Res-Net-50-extracted deep visual aspects are more successful in recognizing nuances, and impulsive micro-signals. Geometric base models tend to overlook these factors.

## 8 Emotion recognition in multimodal mode

MER incorporates text, audio, visual, and physiological inputs to represent emotion more precisely. Each input detects additional emotion-relevant information. Multimodal fusion methods are applied to combine these attributes during modal-dependent attribute extraction. Fusion methods are commonly classified as early, late, and hybrid fusion. Early fusion integrates modal-specific features for dual representation. Late fusion combines the results of multiple modal-specific models. Hybrid fusion utilizes both methods to balance representational diversity and decision flexibility. Such fusion methods enhance robustness contrasted to unimodal models. Still, fusion ability is frequently restricted by temporal misalignment, missing or noisy modalities, modality imbalance, and enhanced computational difficulty Zhang et al. (2022a).

Based on relevant review, the temporal alignment for the input dataset defines the most effective fusion approach. Although early fusion acquires inter-modal connections at the low scale, it performs effectively while different modalities are strongly associated (such as acoustic with facial gestures). Still, when integrating sparse content with complex physical data, it confronts the “curse of dimension.” In opposite, Late fusion is more effective for “in-the-wild” datasets such Aff-Wild2 as it becomes more robust for modality-based noises and data loss. Through dynamically weighting methods, new attention-based transformers have overcome such a gap. It minimizes the severe limitations of typical fusion models.

### 8.1 Model classification

In MER models, classification is the final step after attribute extraction and modal fusion. Fusion methods have been assigned to emotions via both deep learning and traditional classifiers like SVM and RF. Kernel-based SVMs are useful for extremely large emotional features, especially in scarce data contexts, while deep models combine classification and representation learning in an integrated framework. In general, a fully connected layer designed utilizing soft-entropy loss is employed in deep learning models to generate predictions Li and Deng (2020). In contrast, deep classifiers involve large structured databases and can have issues modelling over subjects and modality, while conventional methods depend upon handcrafted characteristics. As a result, classifier selection continues strongly related with data availability, feature structure, and fusion method in MER.

### 8.2 Emotion recognition through audio and visual cues

**FIGURE 7:**
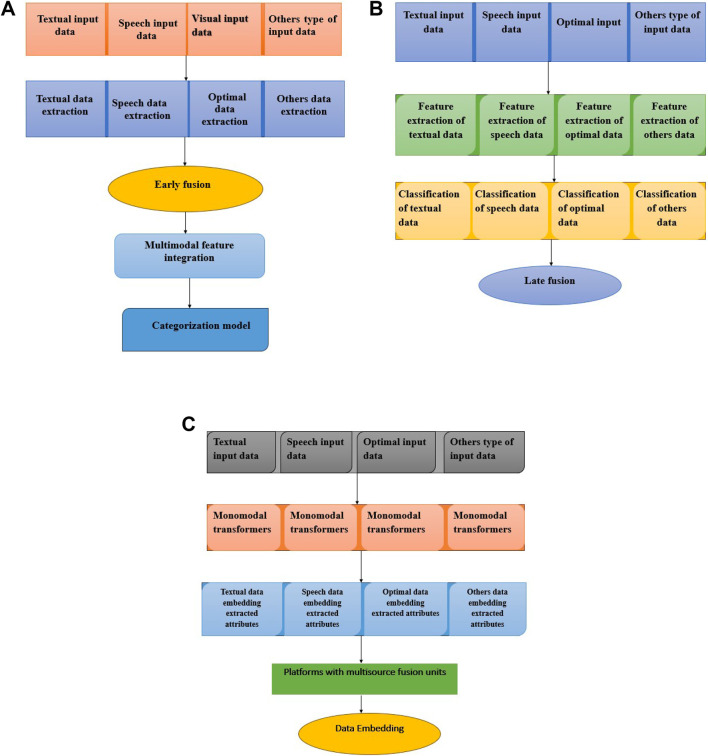
**(A)** Early fusion. **(B)** Late fusion. **(C)** Hybrid fusion.

Although auditory and visual modes are strongly associated with expressing behaviours, both are crucial for MER. Audio features contain spectral and prosodic emotions, whereas visual traits capture facial changes over time. Their fusion yields complementary emotional models and boosts robustness under noise and incomplete settings. Although temporal divergence, sensitivity to occlusion, modal dominance, and audio noise persist to hinder efficient audio-visual fusion, challenging. Figures 7A–C show the pipeline of early, late, and hybrid fusion for emotion recognition Gladys and Vetriselvi (2023). The foundation of our comparison-based differences review is illustrated in Figures 7A–C. By removing the modal superiority isssues that exists in traditional early-fusion methods, hybrid fusion Figure 7C enhances the performance, as illustrated in Table 5. In Table 5 we have shown the audio-visual fusion for bimodal emotion recognition, where we have mentioned the classifier and methods with achieved performance.

**TABLE 5:** Recent work on audio-visual fusion for emotion recognition.

Publication	Dataset	Modalities	Classifiers	Fusion Strategy	Achieved Performance
Nguyen et al. (2018)	eNTERFACE, FABO	A-V	DBN	Early-fusion	90.85% (eNTERFACE); 92.84% (FABO)
Fu et al. (2019)	Enterface ‘05	A-V	SVM	Early-fusion	Acc. Rate: 80.10%
Avots et al. (2019)	SAVEE, eNTERFACE’05, RML, AFEW	A-V	SVM	Hybrid-fusion	Acc.: SAVEE- 94.33%; enterface’05%–48.31%; RML-60.20%; AFEW-94.68%
Zhang et al. (2019)	Video Emotion-8, Ekman-6	A-V	FC with softmax	Early- fusion	8 Classes-54.50%; 6 classes-55.30%
Ghaleb et al. (2019)	eNTERFACE05, CREMA-d	A-V	SVM	Early-fusion	Acc.: 66.5% (eNTERFACE05); 91.5% (CREMA-D)
Huang et al. (2020)	AVEC (2017)	A-V	ANN	Early- fusion	CCC(Arousal): 0.654; CCC(Valence): 0.708%
Hao et al. (2020)	eNTERFACE05	A-V	SVM	Late- fusion	Acc.: 81.36%
Zhao et al. (2020)	CK; AFEW	A-V	Multiple kernel SVM	Early- fusion	7 classes: 95.7%; 7 classes: 45.20%
Krishna and Patil (2020)	eNTERFACE	A-V	Meta-classifier	Late-fusion	6 classes: 81.36%
Farhoudi and Setayeshi (2021)	eNterface’05	A-V	MoBEL	Feature-fusion	Acc.: 81.74%
Sharafi et al. (2022)	SAVEE, RAVDESS, RML	A-V	FC with softmax	Early- fusion	Acc.:SAVEE-99.75%, RAVDESS-94.99%, RML-99.23%
Hsu and Wu (2023)	BAUM-1, CMU-MOSEI	A-V	Bi-modal transformer encoder	Model level fusion	Acc.:74.31% (BAUM-1) Acc.:76.81% (CMU-MOSEI)
Shahzad et al. (2023)	M-LFW, CREMA-D and TESS	A-V	CNN	Early- fusion	Acc.: CREMA-D 75.67%, TESS 99.92%
Kumar et al. (2024)	IIT-R SIER	A-V	CNN	Hybrid- fusion	Acc.: 83.29%
Fan et al. (2024)	RAVDESS, PKU-ER	A-V	MLP	Hybrid-fusion	Acc.:82.62% (RAVDESS); Acc.: 92.60% (PKU-ER)
Hore and Bhattacharya (2025)	RAVDESS + YouTube	A-V	1D-CNN, VGG-face	Late-fusion	Fusion metric outcome: 66%
Sun et al. (2025)	RAVDESS, CREMA-d, CMU-MOSEI	A-V	Dense layer + softmax	Hybrid-fusion	Acc.:97.92% (RAVDESS), 87.20% (CREMA-D), 79.40% (CMU-MOSEI)

The relevant initial studies represent a source of the outcome metrics (Acc. or F1 Score) illustrated in the above table. Due to variations between datasets, evaluation methods, conventional metrics, and subjects, such scores cannot be easily assessed over individual rows. Specific models evaluated in comparable experimental settings can be accurately analysed.

#### 8.2.1 Early fusion

Modal-specific data are collected individually and temporally connected during joint representation learning. Multi-modal emotional interactions are further recorded via feeding the fused feature vector into an integrated learning algorithm. New transformer- and attention-based methods further improve connection among acoustic and visual signals Hossain and Muhammad (2019), Cai et al. (2020), Liu et al. (2025). Early fusion remains vulnerable to temporal mismatch, high-dimensional feature boundaries, and overfitting for inadequate info. Through including modality-dependent variables at the modelling phase, late fusion eliminates the alignment and scalability restrictions of early fusion, which records fine-grained multi-modal correlations across the feature layer.

#### 8.2.2 Late fusion

Late fusion is also referred to as decision-level fusion. Late fusion works well while multiple modalities show temporal mismatch or mode-specific variance. It incorporates decisions using single-mode classifiers instead of integrating features. Thus, retaining modal-specific models decreases alignment cost. Late fusion is frequently utilized through both hard and soft voting, probabilistic inference, and ensemble learning, and is esteemed in its scalability and robustness Salas-Cáceres et al. (2025). Studies with standard databases like RAVDESS and CREMA-D show competitive advantages while retaining structural flexibility. However, in late fusion, inter-modal relationships cannot be directly learnt through representation development. Its effectiveness in modelling intricate emotional patterns can be restricted because of the lack of fine-grained cross-modal interactions.

#### 8.2.3 Hybrid fusion

Hybrid fusion solves the drawbacks of early-level and late-level fusion by fusing shared representations and decision-level reasoning. It preserves adaptability in the appearance of various data settings while enabling fine-grained modal interactions. Representation models, like MCEF (multi-clue emotion fusion), integrates facial geometric, audio signals, and landmarks over fusion levels to improve video-based emotion detection Yan et al. (2016). More new approaches use gated pooling frameworks to boost inter-modal feature interactions. Further, cross-attention methods are applied that represent inter- and intra-modal interactions, showing robustness on complex databases (such as RECOLA, CMU-MOSEI, and Aff-Wild2) Kumar and Aruldoss (2025), Praveen and Alam (2024). Considering their utility, hybrid fusion methods usually involve other computational difficulties. Furthermore, when heterogeneous training data are limited, rigorous layout is essential to avoid overfitting.

### 8.3 Fusion of text, image and speech data

MER incorporates text, audio, visual, and physiological data to record additional emotional data. Unimodal models usually overlook subtle changes in emotions, which promote early, late, and fusion approaches. The most effective approach is hybrid fusion, which represents emotions by integrating common representations and context-sensitive decision models [Jiang et al. (2020a)], Majumder et al. (2019). On standard datasets such as IEMOCAP, new hybrid methods to incorporate transformer-based textual models with deep auditory and spatiotemporal visual traits consistently demonstrate advances Huddar et al. (2021). Still, multimodal fusion continues to be limited by imbalanced data, annotated sparsity, and modality noise. When all factors are evaluated, hybrid fusion presents a scalable and balanced method of multimodal real-life affective computing.

### 8.4 Fusion of multiple physiological signals

Physiological emotion detection systems combine EEG and peripheral data (like EMG, ECG, EDG, PPG, and GSR) with integrated MER systems. Peripheral data are especially sensitive to noisy sensors and motion errors. In the opposite, EEG has inferior ratios of signal to noise with high inter-subject variability but provides immediate neural correlations of emotions. Therefore, finding a fusion method is crucial to predict emotions accurately. Decision-level and hybrid fusion methods demonstrate robustness through concurrently modelling shared and modal-specific representations Hassan et al. (2019a), Zhang et al. (2020b). Recent investigations increasingly employ additional methods, like visual clues and eye-tracking. These signals increase system reliability through adjusting for inconsistent physiological signals Guo et al. (2024). But subject variation and sensor reliance still prevent physiological multisensory fusion. Scalability and real-world application are made more difficult with insufficient data. Multimodal fusion methods combine multiple data sources based on these modal-specific features and drawbacks. Textual, audio, visual, and physiological signals are fused to generate more reliable and comprehensive emotion detection.

### 8.5 Other forms of modality fusion

Multimodal fusion effectively increases emotion detection by fusing related emotional signals. CNN-GRU frameworks used for physiological data enable effective emotion recognition via detecting temporal physiological patterns Orynbassar et al. (2025). Representation-level fusion for EEG, visual, and eye-tracking signals illustrates the advantages of learning multi-modal relationships Wang et al. (2021), Jiang et al. (2022). Audio-visual systems using attention-based CNN-GRU models work effectively on standard databases (like IEMOCAP and RAVDESS) Jia et al. (2022). By modelling temporal emotion evolution, memory-based hybrid systems boost speech emotion recognition significantly Lian et al. (2019). Although multimodal fusion methods continue to be computationally complex and subject to database errors. Their performance usually decreases with missing input and insufficient training data, reducing real-world applications. Other forms of fusion for MER are shown in Table 6.

**TABLE 6:** Recent work on multimodal fusion for emotion recognition.

Publication	Dataset	Modalities	Classifiers	Fusion strategy	Achieved performance
Gao et al. (2018)	MNIST, ORL, Caltech101, RMMLand eNT	Writing, face, object, AV	K-NN, LMCCA	Early fusion	Acc.: MNIST:79.96%, ORL:98.00%, Caltech:83.75%
Lakshminarayana et al. (2019)	MMSE	V, EDA, RR, PR	FFN	Early fusion	F1-score: 58.1%
Barbieri et al. (2019)	OMG	ATV	LSTM, GRU + Attention, 1D CNN, 3D CNN	Late fusion	Avg CCC: 17%
Kanjo et al. (2019)	Envbodysens	On-body, motion, env., location	CNN-LSTM	Early fusion	Acc.:94.7%, Prec:92.7%, Rec:95%, F1:94.9%
Jung et al. (2019)	DEAP, MAHNOB-HCI, AMIGOS, DREAMER	V, EEG, ECG, GSR	PCA, LSTM	Early fusion	Acc.:54.22%–62.07%, mean F1:0.29–0.35
Zhang (2020)	DEAP, DECAF	MP	ANN	Feature-level fusion	Acc.: 69.6%–71.9%
Zhang et al. (2020a)	DEAP	MP	KNN, RF, CART (bagging)	Late fusion	Acc.:94.02%–94.22%
Pei et al. (2020)	RECOLA, AVEC2015	AV, gender	LSTM	Early fusion	CCC:72.7%, RMSE (Val/Aro):82%–99%
Lian et al. (2021)	IEMOCAP, MELD	A + T	ANN, SVM	Early fusion	Acc.:78.08%, 56.67%
Mittal et al. (2021)	IEMOCAP, CMU-MOSEI	A + V + T	ANN	Hybrid fusion	Acc.:78.2%
Antoniadis et al. (2021)	Aff-Wild2	A + V + BM	ANN	Early fusion	Acc.:66.8%
Zhang et al. (2021b)	DEAP, MAHNOB-HCI	EEG, PPS	HFCNN	Early fusion	Acc.:84.71%–89%
Yin et al. (2022)	DEAP, AMIGOS, PMEmo	EDA, music	1D CNN + attention, ResNet18	Early fusion	Acc.:64.5%–83.4%
Wu et al. (2022)	SEED, SEED-V, DEAP	EEG, eye gaze	Deep CCA, SVM	Early fusion	Acc.:84.5%–95.08%
Zhang et al. (2022b)	DEAP, MAHNOB-HCI	EEG, PPS	CNN + MFB	Early fusion	Acc.:90.17%–91.84%
Wang et al. (2023)	DEAP, MAHNOB-HCI	EEG, visual	CNN, dense	Early fusion	Acc.:96.26%–97.15%
Lin et al. (2023)	CMU-MOSEI	A + V + T	FC	Early fusion	Acc.:82.7%–89%
Wang et al. (2022a)	ECF	A + V + T	openSMILE + C3D BiLSTM	Feature-level fusion	F1:0.2581–0.7648
Sharma et al. (2024)	MOOD	V + T	ALFRED + ResNet50 + BERT	Hybrid fusion	F1:66.66%
Gong et al. (2024)	DEAP, DREAMER	EEG, PPS	FC + softmax	Hybrid fusion	Acc.:97.97%–99.47%
Zhang et al. (2024)	RAVDESS	Face, audio	CNN-LSTM	Early fusion	Weighted Acc.:79.40%
Alam et al. (2025)	SEED-IV	EEG + audio	Attention model	Early fusion	Acc.:88.70%
Pillalamarri and Shanmugam (2025)	WESAD	HR, accel, EDA	Ensemble CNN-GRU	Late fusion	Acc.:84.50%

The relevant initial studies represent a source of the outcome metrics (Acc. or F1 Score) illustrated in the above table. Due to variations between datasets, evaluation methods, conventional metrics, and subjects, such scores cannot be easily assessed over individual rows. Specific models evaluated in comparable experimental settings can be accurately analysed.

### 8.6 Discussion

This study provides a valid, data-based structure to present MER. The reliability across textual, acoustic, visual, and physiological data and each provide different emotional features. Feature extraction models increase classification; they are still weak to subject variation, domain transfer, and distortion. Early fusion detects basic connection, but dimensionality, alignment, and insufficient data create difficulties. Deep multimodal processing is reduced for late fusion, while robustness with missing modality is enhanced. Hybrid fusion allows balanced connectivity but improves system intricacy as well as processing complexity. Since they primarily depend on the advanced learning models, such as self-supervised learning, the attention method, transformers, and data quality, which improves alignment. The main obstacles tend to exist data limitations like class imbalance, data scarcity, and acted emotion. Physiological data are affected by subjective variance or sensory distortion, yet they contain hidden emotional data. Generally, combined optimization for fusion model, representation learning, and data quality is essential for effective MER. Individual model enhances individually are inadequate to attain accurate and versatile ER.

A significant effect of experimental approaches on published outcomes is illustrated using a thorough review of performance metrics provided in Tables 5, 6. Since the performance in modern MER systems is high, the basic data-oriented architecture provides a significant effect on the results. In this regard, subjects-independent as well as “cross-subject” reviews performed on “in-the-wild” dataset such Aff-Wild2 usually yield less-effective results over subject-specific analyses, and making them prevalent for lab-based dataset such as SEED. In addition, developing a uniform benchmark becomes difficult because of a shortage of standard training and testing split over several studies. As a conclusion, the overview outcomes values represent an advanced level for certain contexts.

#### 8.6.1 Overview of quantitative insights

Transformer-based hybrid fusion usually exceeds classical early and late fusion via an overall range of 5%–8% in performance (accuracy rate), based on our review of existing MER methods. This trend is observed across datasets such as CMU-MOSEI and IEMOCAP. Such a pattern can be presented in dataset such IEMOCAP and CMU-MOSEI. In addition, the combination of eye movement and EEG can enhance affective clarity through around 9%, based on quantitative outcomes from physiological data (like DEAP, SEED). This enhancement is noticed contrasted to single-mode EEG models. These outcomes illustrate the reality that cross-modal attention mechanisms are the cause of the substantial enhancement in performance within existing MER studies. Sequential data sources are successfully handled via these approaches.

#### 8.6.2 Comparative performance: self-supervised vs. supervised approaches for MER

There are several significant differences between supervised learning and self-supervised learning approaches for MER, based on a comprehensive review of existing studies. High accuracy is presented by using supervised-based learning models, which are employed in the early fusion models with the SEED and IEMOCAP data. Still, these methods are fundamentally restricted with the shortage of high-quality, classified heterogeneous data and the “attention bottleneck.” In opposite, SSL generates effective, context-specific models by leveraging extensive unlabelled corpus. This substantially enhances efficiency in data scarcity contexts. Based on quantitative studies, SSL-related pretraining like BERT for textual and Wav2Vec for acoustic data demonstrates superior subject adaptability over traditional supervised approaches. These traditional approaches usually struggle from overfitting issues on limited datasets. Such limitation becomes most prominent in unbalanced datasets [like RECOLA (Praveen et al., 2021)]. Self-Supervised Learning frameworks provide higher “Ecological Validity,” while supervised models execute effectively in limited, lab-based applications. Whenever they transfer towards “in-the-wild” environment, overall F1-scores are still constant. Labelled data are substantially insufficient for these environments. As an outcome, the transition to SSL constitutes an appropriate response to the data-oriented issues determined in this study.

### 8.7 Unified evaluation model for MER

This review shows a unified review process to solve the existing scarcity of standards over textual, acoustic, visual, and physical approaches. It integrates data-centric drawbacks and performance metrics. Using this architecture, the primary focus is changed from high efficiency into a model’s potential to resist biases as well as distortion related for an individual mode. Our review reveals that high-quality time synchronization is needed for biological signals like EEG. In opposite, robustness over brightness and posing shifts is crucial for “in-the-wild” visual information. This concept determines systems’ abilities to extend over various contexts via classifying those due to the concept of ecological validity. The capacity to preserve significant F1-values and shifting between lab-based datasets such as SEED to unlimited contexts such Aff-Wild2 is defined as ecological validity. The obstacles of shifting between restricted to real-life settings are demonstrated through this gap. Therefore, the system presents a consistent standard to determine real model scalability in cognitive computing scenarios.

### 8.8 Handling misssing modalities in MER

Our data-specific review demonstrated that missing modality throughout traditional inference tend to be a significant barrier for real-world application. Several fundamental approaches are utilized in current studies to ensure adaptability for the lack of data sources (like distorted EEG or restricted face-based videos):

#### 8.8.1 Cross-modal recovery

Reconstructive focuses and unified layers are incorporated by models like adaptable audio-visual learning to mask feature gaps. As an example, the framework restores the latent model for the missing face attributes with the audio embedding approach whenever an optical input is destroyed. This helps ensures superior performance for datasets (such as CMU-MOSEI).

#### 8.8.2 Unified feature pretraining

With the goal to develop a unified semantics domain, self-supervised algorithms pretrain frameworks on large amount of unstructured data. This facilitates the system to assign a multi-dimensional emotions structure into sparse data, such text alone. Primarily, this pattern was constructed with entire multimodal data (such as textual, acoustic, and visual data).

#### 8.8.3 Gated attention fusion mechanism

Advanced hybrid models continuously adjust for modalities relevance via gated pooling and cross-attention approaches. Such gates basically zero out particular modality’s impact whenever it is defined as “missing” and “noise”. This removes the requirement of an extensive structural review and allows the framework to depend entirely on the present consistent sources.

Transformer-based models present the most effective solution for data shortages, which can be illustrated by sorting scholarly articles due to these specific approaches. These approaches perform more efficiently than classical early fusion approaches. Insufficient data vectors tend to cause such conventional methods to be ineffective.

## 9 Limitations

Scarcity and heterogeneity of Multimodal Data: An obstacle of contemporary MER study is the insufficient accessibility of vast and heterogeneous multimodal datasets. Maximum datasets which are available publicly, incorporate a several number of topics. This existing data is based on acted expressions, which may not fully reflect actual emotional states. It is very costly and laborious to collect aligned data over textual, verbal, facial gestures and bio signals, specifically when physiological data are intricated. Same standard datasets are used in many research, as a result leading to dataset reliance and limited conception. Additionally, divergence in interpretation systems, emotion frameworks and capturing situations makes it hard to equate outcomes over research. These dataset obstacles limit the reliability and application of MER model.

Data Intricacy and Imbalanced data: From imbalanced data and the intricacy of emotional interpretation, one other challenge emerges in multimodal data. Happy and neutral modes of emotions are arising often that control maximum datasets, and fear, confusion, or disgust are infrequent modes of emotions that are marginal. This imbalanced data skews learning algorithms to the upper class and decreases the performance of recognition for low-frequency emotions. Furthermore, emotions vary over interpreters, circumstances and cultures, and emotional interpretation is fundamentally subjective. Emotional states are not shortly visible and need expert annotation. Furthermore, biosignal data involve interpretation. These problems undermine training scalability and decrease the durability of performance metrics like accuracy and F1-score.

Distortion, Disturbances, and Deterioration: Over modalities, MER models are hypersensitive to distortion and deterioration. Audio data are frequently simulated with background distortion, recording environment, and variability of the speaker. Facial gestures abide by light transition, obstruction and head pose changes. Biosignals like ECG, EEG, and EMG are unusually sensitive to distortion, lead misplacement, and inconsistency. Preprocessing and attribute extraction are remarkably simulated by these annoyances. As a result, inconsistent emotional expressions arise. Even though noise-reduction methods are applied, they are data-centric and cannot simplify environments well. Furthermore, signal quality endures as a crucial issue for reliable and powerful emotion recognition.

Missing Modality and Real-World Deficiency: Maximum MER research affects the accessibility of all modalities through training and testing; that is an unusual case in everyday usage. In real-world circumstances, modalities can be missed because of sensor defaults, privacy risks, or user resistance. For instance, sometimes biosignal sensors may be wearable, or facial gestures may be inaccessible because of obstruction or camera restrictions. Available and standard datasets usually model such deficient circumstances. These make an aperture between study and implementation. Models trained under a complete hypothesis often stop when faced with limited or real-time data. This obstacle decreases the relevance of MER systems in reality, like man-machine interaction, medical care, and education.

Interdisciplinary and Multi-Domain Concept Issues: Emotional expression remarkably changes among individuals because of culture, personality, gender, age, and biosignal features. Models trained on restricted topics frequently perform poorly when tested on anonymous users, specifically for biosignals that are extremely dependent on the subject. Additionally, MER systems trained with limited datasets fight to extend to real-world environments like digital environments, medical supervision, and communication systems. Domain transfer is affected by variations in syntax, emotional environment, sensation level, and external variables. The majority of research does not appropriately address inter-subject or multi-domain durability and prefers to focus on across-dataset review. Therefore, several MER systems’ ability for extension is still hindered.

### 9.1 Strategic roadmap and solutions

Although the technical difficulties related to data insufficiency as well as class imbalance have been explored previously, the current section presents particular, practical approaches to the above obstacles. These approaches have been developed in MER models for the future.

Domain adaptation and zero shot learning researchers should employ UDA (unsupervised domain adaptation) to tackle the subject-based biases described previously. It enables the extension of an approach trained on an extensive lab-controlled datasets (such as SEED) over new, unstructured real-life subjects. It achieves these without the necessity of significant new remarks.

Weakly supervision-based and self-supervised representation learning MER models must focus on self-supervised pretraining on enormous unstructured datasets to enable to minimize high-annotation costs. Tiny, reliable emotive datasets are then usable to enhance such models. This minimizes the system’s requirement for precisely balanced classes by leveraging global patterns.

Federated Learning for protected physiological signals confidentiality is a significant challenge in the acquisition of physiological signal. We propose utilizing Federated Learning, by which individual (like wristbands and EEG headbands) are employed for training systems locally. It preserves essential basic physical signals confidential and enhancing data size as well as data variation.

Data synthesis and augmentation future studies must go over conventional noise input to solve data limitations. Leveraging GANs (Generative Adversarial Networks) and Diffusion Models to develop exceptional emotions such severe disgust or fear are feasible solutions. This supports to balance unbalanced data like Aff-Wild2.

Finally, adaptable data-based approach needs to substitute model-based intricacy in the actual research objective for the future version of MER. The development of CMR (Cross-Modal Reconstruction) models needs to be the primary goal of research. These models hallucinate data loss sources via transformer-based distributed latent areas. Such as, researchers can utilize audio prosody to reproduce facial nuances. By concentrating on distributed self-supervised learning, the domain may prevent the cost as well as confidentiality difficulties posed by acquiring large amounts of physiological data. This method makes it feasible to develop trustworthy systems. Such models are able of being used effectively within unlimited, “in-the-wild” contexts.

## 10 Conclusion and future direction

### 10.1 Conclusion

This study shows how advanced MER models have developed. Still, the lack of standard “Resilience Metrics” generates an actual gap. Performance on extensive datasets is the primary objective for existing studies. Though, research analysing whether simulations sustain affective fidelity is notably limited. This represents particularly apparent if several modes (such as optical or EEG) become sequentially deferred and gradually eliminated via immediate interpretation. Moreover, there remains a significant gap for subject-specific domain adaptability. Due to lack of substantial reconstitution, a large number of advanced models for physical data, including these employing DEAP or SEED, struggle to predict over a range of users. This constraint is a substantial challenge to practical scalability. This study has shown a complete, modality-based evaluation of MER emphasis on types of data, standard datasets, preprocessing methods, and attribute extraction approaches. By an organised review of text, audio, visual, and biosignal data, this study illustrates the importance of data integrity in predicting the efficiency of MER and shows that each medium transmits useful emotional data. This study serves as an essential resource to scholars aiming to build more reliable, versatile, and human-friendly MER models by integrating signal-related data among mediums and identifying unknown future possibilities. The integrity, variety, and accessibility of emotions utilised by advanced learning will remain essential to MER’s future improvement. The essential review reveals that instead of lacking effective learning models, existing MER challenges are mainly driven by insufficient datasets, noisy and missing data, and unsatisfactory adaptation over subjects and contexts. Transitioning of data-based studies that highlight database variation, reliability, and real assessments is required to solve these problems.

### 10.2 Future direction

Further studies must require data-driven, robustness-based approaches to extend MER over limited standards. First, it is important to generate enormous, wide-range, and consistent multidimensional datasets. Current datasets will enable all unimodal and multimodal emotion models to collect real emotions in a number of languages, circumstances, and cultures. An appropriate benchmark dataset requires resolving imbalances in class with balanced data observation or semantic methods of assessment. Second, one of the primary objectives of the study must be to deal with distorted and lacking modality effectively. Later MER models should be designed for use with insufficient, weakened, or inconsistent data instead of assuming accurate data accessibility. In a real-world environment, uncertain evaluation approaches and neutral and data-driven production processes will boost system dependability. Third, larger amounts of data and assessment approaches are necessary to enhance domain-wide adaptation. In terms of real-world use, training and testing methods must especially take subject-specific and diverse data variables into account. Systems will develop into emotion-free models over data-centric features if datasets from various domains and different regions are assembled. In conclusion, both the data and attribute-level accessibility and transparency must be enhanced. Reliability requires recognizing whose modality and features are essential to recognize emotions, particularly for critical situations such as the medical field and education. By illuminating the encoding of emotions over different modes, data-oriented explanation may enhance model-specific interpretation. The comprehensive usage of LLMs represents a potential solution for overcoming a shortage of reliable multi-modal data. LLMs can be implemented in synthesised data extraction to enhance minority classes for imbalanced datasets, as well as to its existing use for defining features. As an example, generative models generate text or aural translations which are comprised of contextual information and diversity. Emotional intricacies are retained within these translations. Without the costly nature of individual annotation processes, this approach rapidly expands the training corpora. In addition, fundamental frameworks’ zero-shot and few-shot transfer learning features allow MER models to detect emotions in scarce languages or novel areas without the requirement of significant task-oriented labelling. Modern MER models allow for high efficiency even with the presence of significant data limits by utilizing the wide-ranging global data pre-trained within such models.
